# Crumbling the Castle: Targeting DNABII Proteins for Collapsing Bacterial Biofilms as a Therapeutic Approach to Treat Disease and Combat Antimicrobial Resistance

**DOI:** 10.3390/antibiotics11010104

**Published:** 2022-01-14

**Authors:** James V. Rogers, Veronica L. Hall, Charles C. McOsker

**Affiliations:** Clarametyx Biosciences, Inc., 1275 Kinnear Rd, Columbus, OH 43212, USA; jrogers@clarametyx.com (J.V.R.); vhall@clarametyx.com (V.L.H.)

**Keywords:** antibiotic, antimicrobial resistance, biofilm, DNABII, histone-like protein (HU), integration host factor (IHF)

## Abstract

Antimicrobial resistance (AMR) is a concerning global threat that, if not addressed, could lead to increases in morbidity and mortality, coupled with societal and financial burdens. The emergence of AMR bacteria can be attributed, in part, to the decreased development of new antibiotics, increased misuse and overuse of existing antibiotics, and inadequate treatment options for biofilms formed during bacterial infections. Biofilms are complex microbiomes enshrouded in a self-produced extracellular polymeric substance (EPS) that is a primary defense mechanism of the resident microorganisms against antimicrobial agents and the host immune system. In addition to the physical protective EPS barrier, biofilm-resident bacteria exhibit tolerance mechanisms enabling persistence and the establishment of recurrent infections. As current antibiotics and therapeutics are becoming less effective in combating AMR, new innovative technologies are needed to address the growing AMR threat. This perspective article highlights such a product, CMTX-101, a humanized monoclonal antibody that targets a universal component of bacterial biofilms, leading to pathogen-agnostic rapid biofilm collapse and engaging three modes of action—the sensitization of bacteria to antibiotics, host immune enablement, and the suppression of site-specific tissue inflammation. CMTX-101 is a new tool used to enhance the effectiveness of existing, relatively inexpensive first-line antibiotics to fight infections while promoting antimicrobial stewardship.

## 1. Introduction

Antibiotics revolutionized the global healthcare industry by ushering in an era of significantly lower morbidity and mortality associated with lethal bacterial infections. However, many bacterial species have developed one or more mechanisms of resistance to almost every antibiotic developed since the discovery and introduction of penicillin [1], causing the World Health Organization (WHO) to declare antimicrobial-resistant (AMR) bacteria a top ten global health challenge threatening the world today [2]. According to the Centers for Disease Control and Prevention (CDC), the United States has more than 2.8 million antibiotic-resistant infections annually, leading to more than 35,000 deaths; the misuse and overuse of antimicrobial agents are the two main drivers of the development of AMR bacteria [3]. The clinical outcomes associated with AMR are increased morbidity/mortality, longer hospital stays, and substantial healthcare and societal costs. Nelson and colleagues attributed annual US hospital charges of USD 4.6 billion to multidrug-resistant infections due to only six pathogens [4]. Similar health and economic impacts have been estimated by the European Medicines Agency (EMA) and the European Centre for Disease Prevention and Control (ECDC) [5]. By 2050, it has been projected that deaths due to AMR could rise to 10 million lives each year, with a cumulative global cost of USD 100 trillion, if significant action in combatting AMR is not taken [6]; however, while not questioning the severity of the AMR problem, the methodologies used to arrive at these estimates have been questioned [7].

A compounding factor in the increasing prevalence of AMR bacteria is that the discovery and development of new antibiotic classes have been essentially stagnant for decades, which has limited the options for physicians to treat AMR bacterial infections. Many large pharmaceutical companies have abandoned developing and producing new antibiotics due not only to the risky and prolonged R&D process, but also to their low profitability due to the generally short duration of therapy and limited market lifetime, competition from generics, and stewardship practices that significantly restrict the use of new antibiotics. In response, worldwide “push” and “pull” initiatives have been implemented to stimulate antibacterial research and development. The Generating Antibiotic Incentives Now (GAIN) Act, which was passed in the US in 2012, exemplifies a “pull” initiative that is intended to drive antibiotic innovation and R&D. Within five years of GAIN Act enactment, the US Food and Drug Administration (FDA) granted 147 Qualified Infectious Disease Product (QIDP) designations, including approximately 74 designations for novel drugs, culminating in the approval of 12 drug products being granted a QIDP designation [8]. One of several successful “push” initiatives is the Combating Antibiotic-Resistant Bacteria Biopharmaceutical Accelerator (CARB-X; www.carb-x.org), a global nonprofit partnership dedicated to accelerating antibacterial research to combat AMR bacteria. As part of the CARB-X portfolio, 22 companies (including Clarametyx Biosciences, Inc.; www.clarametyx.com) are actively conducting research and development of nontraditional therapeutics, such as antivirulence strategies, bacteriophages, and approaches to modulate the microbiome [9].

The rise in AMR is due in part to inadequate treatment options for biofilms formed during bacterial infections. It is well accepted that biofilms are the preferred growth form of bacteria. Biofilms are implicated in 65–80% of microbial infections [10,11,12]. Bacteria encapsulated in biofilms may be up to 1000-fold more tolerant to antibiotics, promoting the evolution of antibiotic resistance by requiring higher antibiotic doses and longer treatment regimens [13,14,15,16]. With the declining effectiveness of existing antibiotics and the lag in the discovery and production of new antibiotic classes, it is imperative to progress new and innovative drug modalities and nontraditional therapeutics through the clinic to backfill the gap created over the past three decades [9]. Many efforts are underway to develop new products and clinical management strategies to combat the growing problems of AMR and nosocomial infections related to biofilms [13,17,18,19,20,21,22].

This article highlights the development of one such novel nontraditional product, CMTX-101, an anti-DNABII humanized monoclonal antibody being developed by Clarametyx Biosciences, Inc. CMTX-101 targets a universal component of bacterial biofilms and causes rapid and pathogen-agnostic biofilm collapse, thus enhancing the effectiveness of innate immune effectors and killing by antibacterial agents, enabling existing, relatively inexpensive first-line antibiotics to fight infections better.

## 2. Bacterial Biofilms and Antimicrobial Resistance

Biofilm formation is a well-orchestrated and dynamic event, leading to a community of microbes with division of labor (e.g., altered gene expression) and intercellular communication (e.g., quorum sensing), as well as the creation of a self-made extracellular polymeric substance (EPS) that enshrouds and protects resident bacteria from the environment, antimicrobial compounds, and host immune effectors [15,23,24,25]. The biofilm EPS is a semi-permeable barrier composed of a variety of biological materials, including extracellular DNA (eDNA), proteins, lipids, polysaccharides, and divalent cations [23,26,27].

**Figure 1:**
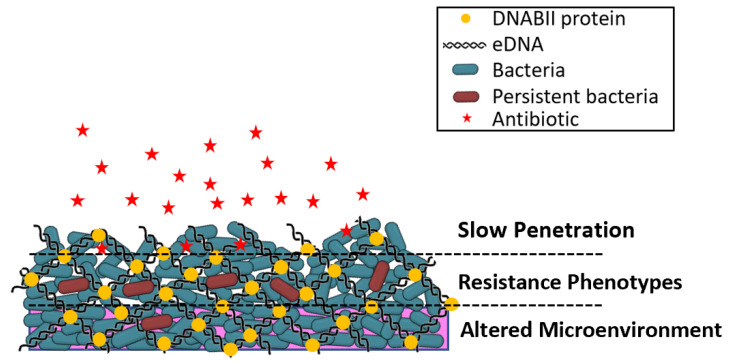
Illustration of biofilm structure in the context of the development of bacterial resistance. Slow penetration—antibiotics (red stars) may fail to penetrate beyond the surface of the biofilm. Resistant phenotypes—bacteria may develop persister cell phenotypes and undergo lateral transfer of genetic resistance elements; bacterial growth rates and metabolic activities are altered from planktonic bacteria. Altered microenvironment—nutrient depletion and accumulation of waste within the biofilm antagonizes the action of antibiotics. Adapted from Reference [32].

Irrespective of the bacterial species that make up the biofilm, bacteria are more antibiotic tolerant within a biofilm. Factors influencing biofilm tolerance following antibiotic exposure include the cell density, the time of treatment, and the age of the biofilm; antibiotic tolerance is not influenced by the molecular weight or the structural class of the antibiotic, the substratum material, or the bacterial species [28]. Biofilm bacteria demonstrate differing characteristics compared to their planktonic (free-living) counterparts, including an increased rate of EPS production, reduced metabolism, altered proteome, and decreased growth rate [16,29]. The regulation of growth through quorum sensing can also promote tolerance to antibiotics, since slow-growing bacteria are generally less susceptible to antibiotics [30]. Additional multifaceted mechanisms attributed to the promotion of antibacterial tolerance include the activation of stress responses due to limited nutrients, restricted penetration of certain antibiotics due to chelation by EPS components, expression of biofilm-specific genes, and the presence of slow-growing persister cells [13,18,25,31]. Figure 1 is a simplified illustration of a biofilm structure in the context of the development of bacterial resistance.

Clinically, the tolerance mechanisms to host immune effectors and antibiotic treatments associated with the biofilms are confounding factors in the effective eradication of the biofilm-resident bacteria. One problem in the treatment of biofilm-related infections with antibiotics is that the dose and treatment regimen for a specific antibiotic type or class is based on data derived from classic antibiotic susceptibility tests, yielding a minimal inhibitory concentration (MIC) value for planktonic bacteria. As biofilm bacteria can be orders of magnitude more tolerant to antibiotic treatment compared to planktonic bacteria, the results of such testing to establish a therapeutic dose may not translate into clinical success in treating recalcitrant biofilm-related infections [18]. Inadequate treatments, without eradication of the persister cells resident within biofilms, can play a role in developing AMR bacteria arising from biofilm-associated infections. Novel approaches are needed to effectively treat bacterial biofilms in order to increase the probability of disease resolution and to decrease the likelihood of AMR development.

## 3. Targeting DNA-Binding Tip Regions of DNABII Proteins to Rapidly Collapse Biofilms

While the molecular makeup of the EPS varies among bacterial species, eDNA is a common component, forming a structural lattice that is critical for biofilm integrity, and is essential for biofilm stability during the early stages of biofilm development [23,33]. In addition to providing structural stability, eDNA exhibits antimicrobial activity and cation chelation, provides a source of phosphorous, carbon, and nitrogen for the resident bacteria, facilitates horizontal gene transfer, induces antibiotic resistance, and contributes to interstitial biofilm expansion [34,35,36,37,38]. As a universal component of biofilms, eDNA is a potential target for disrupting biofilms. DNase treatment can prevent biofilm formation and disrupt immature biofilms by degrading the eDNA lattice. However, DNase treatment does not effectively treat mature biofilms despite the abundance of eDNA [23,33,39]. Recent data show that resistance to nuclease-mediated degradation appears to be due to the accumulation of Z-form DNA within the EPS of mature biofilms, which enhances biofilm structural integrity through stabilization by DNABII proteins [40].

DNABII proteins are a family of bacterial DNA-binding proteins that play a critical intracellular role in bacterial DNA replication and have an essential extracellular role in biofilm formation, stabilization, and maturation. There are two members of the DNABII protein family: integration host factor (IHF) and histone-like protein (HU). These are ubiquitously expressed among eubacteria and do not have any known homologs in mammalian species. IHF and HU generally have a conserved amino acid sequence homology in the DNA-binding region, and perhaps more importantly, a highly conserved three-dimensional conformation that enables the DNABII proteins to bind with high affinity to the pre-bent Holliday junction-like structures that exist in the eDNA lattice of bacterial biofilms [41,42]. Table 1 demonstrates that the primary amino acid sequence of DNABII proteins in the DNA-binding region is generally conserved across bacterial pathogens. This is not surprising considering that the function of DNABII proteins is to bind and bend DNA to support bacterial replication and eDNA lattice formation. It is speculated that those bacterial species with a lower degree of primary sequence homology in their DNABII proteins still have a conserved secondary structure that enables the DNABII proteins to bind and bend DNA. The ability of anti-DNABII antibodies to disrupt biofilms even from species with a lower degree of primary sequence homology suggests the targeting of such a conformational epitope. DNABII proteins serve as linchpins positioned at the vertices of crossed eDNA strands within the biofilm matrix, thereby contributing to the structural stability of the biofilm matrix [39,43,44,45,46,47]. The removal of DNABII proteins from the biofilm destabilizes the eDNA structural lattice and results in the rapid collapse of the biofilm [45]. The conserved DNA-binding region of DNABII proteins and its critical role in biofilm stabilization makes it an ideal target for pathogen-agnostic biofilm disruption.

**Figure 2:**
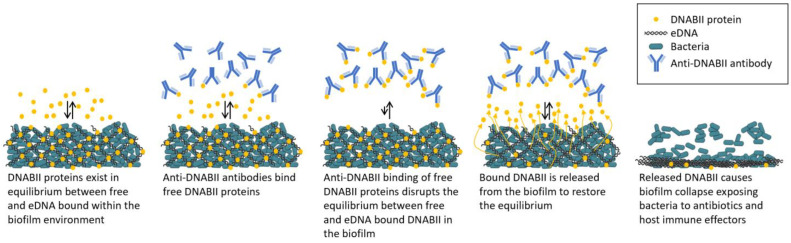
Working model of biofilm collapse following treatment with anti-DNABII antibodies. Adapted from Reference [49].

Anti-DNABII-antibody-induced biofilm collapse does not require physical contact with the biofilm [49]. A working model [49] (Figure 2) for the mechanism by which anti-DNABII antibodies disrupt bacterial biofilms suggests that the exposure of biofilms to anti-DNABII antibodies induces an equilibrium shift between the DNABII protein molecules bound to the eDNA within a biofilm (“on”) and those in an “off” state. DNABII protein molecules in the “off” state are sequestered by anti-DNABII antibodies, forcing bound (“on”) DNABII protein molecules to dissociate from the biofilm eDNA and inducing the structural collapse of the biofilm. This mechanism suggests the potential for targeting DNABII proteins with an anti-DNABII antibody for the treatment of diseases with a biofilm component.

In support of the mechanism described above, antisera generated against IHF complexed to DNA did not collapse biofilms, whereas antisera generated against native IHF rapidly collapsed biofilms [49]. Epitope mapping of IHF identified the DNA-binding region of the DNABII protein as the target of anti-DNABII antibodies that caused biofilm collapse [49]. Monoclonal antibodies targeting the α- and β-subunit DNA-binding tip regions, alone and in combination, effectively collapsed biofilms [50]. Highly potent monoclonal antibodies generated from a chimeric peptide antigen comprising the α- and β-subunit DNA-binding tip regions joined via a short flexible linker (tip chimer) were similarly shown to cause rapid biofilm collapse [48]. The humanization of a monoclonal antibody capable of binding the tip chimer provided the lead compound, CMTX-101.

The rapid collapse of biofilms using anti-DNABII antibodies has been demonstrated in more than 20 Gram-positive and -negative bacterial species in vitro [39,43,45,48,49,51,52,53,54,55,56,57] and in vivo [48,50,55,58,59], as well as in polymicrobial biofilms from clinical specimens [46,60]. Table 2 lists the bacterial species and strains tested to date. Anti-DNABII antibody treatment leads to a significant decrease in biofilm biomass within 5 min [48]. Bacteria released from biofilms upon rapid collapse have been referred to as Newly Released (NRel) and show a transient phenotype that is proteomically and transcriptionally distinct from planktonic bacteria and from bacteria released by slow dispersal [54]. Using an acute otitis media model in chinchillas challenged with nontypeable *Haemophilus influenzae* (NTHI), NRel bacteria generated upon rapid biofilm collapse showed an increased expression of genes associated with cell envelope biogenesis, translation, and ribosomal structure and biogenesis, consistent with the observed sensitization to amoxicillin-clavulanate [54]. The transient and unique phenotype of NRel bacteria from rapidly collapsed biofilms accounts for previous in vitro [39,45,49,51,53,54,61] and in vivo [50,59] observations in multiple bacterial species of enhanced killing by common first-line antibiotics when used in combination with anti-DNABII antibodies. Although the degree of sensitization of NRel bacteria to different classes of antibiotics may differ, we have observed no instances where NRel bacteria are less sensitive than planktonic bacteria.

## 4. Tripartite Mode of Action

**Figure 3:**
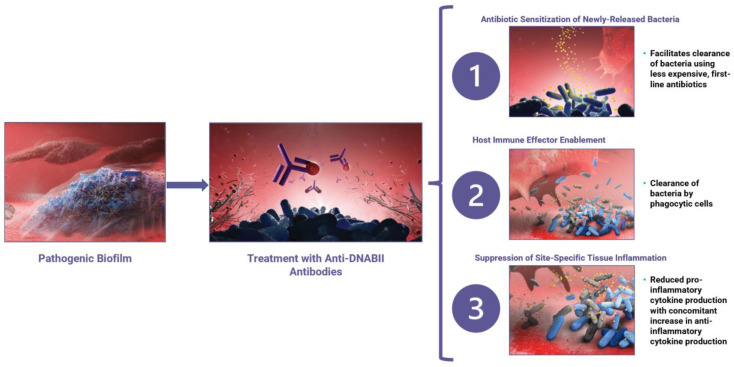
Treatment of an established pathogenic biofilm (left panel) with anti-DNABII antibodies (purple; middle panel) leads to the capture and removal of available key linchpin DNABII proteins (red; middle panel) from the extracellular matrix, resulting in rapid biofilm collapse. This rapid collapse leads to three distinct modes of action (right panel): (1) sensitization of bacteria to antibiotics, (2) host immune system enablement, and (3) suppression of site-specific tissue inflammation.

Anti-DNABII-antibody-mediated rapid biofilm collapse engages several modes of action against both Gram-positive and Gram-negative bacteria (Figure 3). These activities include: (1) the sensitization of released bacteria to the activity of multiple classes of antibiotics; (2) the enablement of bacterial clearance by the innate immune system; and (3) the establishment of an anti-inflammatory environment that is characterized by both inhibition of pro-inflammatory cytokine release and stimulation of anti-inflammatory cytokine release.

### 4.1. Mode of Action 1: Anti-DNABII-Antibody-Induced Rapid Biofilm Collapse Sensitizes Bacteria to Antibiotics

The incubation of preformed bacterial biofilms with anti-DNABII antibodies sensitizes NRels to antibiotic action, resulting in increased antibacterial activity. For example, the treatment of biofilms with anti-DNABII antibodies in the presence of ampicillin, amoxicillin-clavulanate, or cefdinir demonstrated four- to eight-fold decreases in the bacterial minimal inhibitory concentration (MIC) compared to the measured MIC for planktonic bacteria in vitro [49]. Sensitization to antibiotics was also observed in a murine pulmonary infection model with *P. aeruginosa* treated with anti-DNABII antibodies and tobramycin; anti-DNABII antibody treatment was not only effective in reducing lung CFU burden when administered by itself, but also further reduced CFU burden when co-administered with tobramycin [50]. Furthermore, Fab fragments generated from a humanized anti-DNABII monoclonal antibody in combination with ofloxacin eradicated NTHI and cleared biofilms from the middle ear after a shortened course of treatment [59].

### 4.2. Mode of Action 2: Anti-DNABII-Antibody-Induced Rapid Biofilm Collapse Enhances Innate Immune System Clearance of Bacteria

Bacteria in biofilms are generally protected from clearance mediated by the innate immune system (neutrophils, macrophages). The anti-DNABII antibody treatment of biofilm-mediated infections in murine lung, chinchilla ear, and rat oral cavity enables complete or nearly complete resolution in the absence of antibiotic co-administration [50,55,58,59], demonstrating that antibody-mediated biofilm collapse enables more effective neutrophil/macrophage-mediated bacterial clearance. An analysis of lung tissue after antibody treatment also showed a decrease in the number of bacterial aggregates and the recruitment of neutrophils to sites of bacterial aggregates in the lung [50]. Moreover, the intravenous administration of CMTX-101 alone significantly increased survival in a lethal mouse pulmonary challenge model (Clarametyx Biosciences, unpublished data).

### 4.3. Mode of Action 3: Anti-DNABII Antibody Treatment Promotes Establishment of an Anti-Inflammatory State

In a chinchilla model of otitis media using NTHI, the administration of 1 or 2 doses of a humanized anti-DNABII monoclonal antibody directly to the middle ear rapidly cleared the biofilm without the administration of an antibiotic [55]. When compared to controls, anti-DNABII antibody administration and the subsequent biofilm clearance was accompanied by a significant reduction in pro-inflammatory cytokines and a concomitant significant increase in anti-inflammatory cytokines in the middle ear fluid. This phenomenon has not yet been examined in other in vivo infection settings, but may represent a third mode of action by which the host milieu is modified to enable further engagement of the innate immune effectors initially attracted to the infection site by the presence of inflammatory cytokines.

## 5. Clinical Benefits of Anti-DNABII Antibodies

Infections attributed to bacterial biofilms can be separated into device- and tissue-associated infections [63]; examples of device-associated biofilm infections include infections associated with neurosurgical implants, orthopedic implants, artificial heart valves, pacemakers, and vascular catheters, while tissue-associated biofilm infections can include chronic urinary tract infections, chronic wound infections, ventilator-associated pneumonia, chronic rhinosinusitis, osteomyelitis, otitis media, and periodontitis. Due to their high tolerance toward antibiotics and evasion of innate immune surveillance, biofilm bacteria associated with device- and tissue-related infections are difficult to treat, leading to the potential for chronic infections, resistance development, and mortality in patients. Antibiotic treatment and innate immune effectors can usually readily remove bacteria not associated with biofilms; however, the bacteria within biofilms often survive antibiotic treatment, leading to recurrent infections. The failure of antibiotic treatment may necessitate the removal of an implanted device or the surgical excision of infected tissue; therefore, biofilm tolerance to antibiotics directly impacts patient-management strategies and overall clinical outcome [13,64,65,66]. To date, no clinical data on anti-DNABII antibodies have been reported.

## 6. CMTX-101: A Nontraditional Treatment for Attacking Bacterial Biofilms

The high prevalence of biofilm-associated infections and the mechanism by which the anti-DNABII antibody, CMTX-101, collapses biofilms across many species uniquely positions this therapeutic modality for treating a wide variety of biofilm-related bacterial infections. The use of CMTX-101 as an adjunctive therapy to the antibiotic standard-of-care has the potential to increase the effectiveness of the currently moderately effective first-line antibiotics, reducing the need for newer second- or third-line antibiotics, and thus promoting antibiotic stewardship. Since antibiotic resistance may be promoted within bacterial biofilms through mechanisms such as horizontal gene transfer, effective biofilm collapse through the action of CMTX-101 may reduce the potential for bacterial resistance development through such mechanisms. Antimicrobial resistance is a well-documented global issue with significant short- and long-term costs and implications; CMTX-101 is a transformational therapeutic designed to combat AMR through its novel mechanism of action, enabling shorter and more effective antibiotic therapy regimens while improving overall patient outcomes.

As AMR continues to grow as a major worldwide health problem, antibiotic stewardship is assuming increasing importance. Stewardship programs include approaches to promote the appropriate use of antibacterial agents, improve patient outcomes, reduce microbial resistance, and decrease the spread of infections caused by multidrug-resistant organisms. Through its proposed tripartite mechanism of action, CMTX-101 affords a novel approach to addressing AMR, thereby supporting antimicrobial stewardship goals and helping to reduce AMR:

- **Promote the appropriate use of antibacterial agents**: When CMTX-101 disrupts biofilms, NRel bacteria are released with increased sensitivity to multiple antibiotic classes. This enables the effective use of older antibacterial agents, whose use is currently limited by AMR, and reduces the pressure to use newer antibacterial agents as first- or second-line therapies.
- **Improve patient outcomes**: Biofilms reduce the access of antibacterial agents, as well as macrophages and neutrophils, to bacteria; thus, treatment generally requires higher doses and longer courses of antibiotic therapy to achieve a clinical cure. CMTX-101-mediated biofilm collapse sensitizes bacteria to antibiotic action, enhances bacterial clearance by neutrophils and macrophages, and promotes the establishment of an anti-inflammatory environment, increasing the likelihood of improved outcomes when treating bacterial biofilm infections and enabling shorter courses of antibacterial therapy.
- **Reduce microbial resistance**: The mechanism by which CMTX-101 disrupts biofilms suggests a low potential for resistance development due to two factors. First, the DNABII proteins are the only nucleoid-associated proteins that can stabilize the biofilm [52]; however, they are also essential proteins for intracellular DNA-processing where mutations in *ihf* or *hu* alleles severely reduce bacterial fitness [67,68]. Second, the mechanism of biofilm disruption by CMTX-101 does not exert direct selective pressure on bacterial survival, further reducing the probability of resistance development.
- **Decrease the spread of infections caused by multidrug-resistant organisms**: CMTX-101 is active against biofilms formed by all bacterial pathogens examined to date, including ESKAPEE and multidrug-resistant (MDR) pathogens. We have specifically observed that biofilms formed from MRSA and tobramycin-resistant *P. aeruginosa* are rapidly disrupted by CMTX-101 (unpublished data). NRel bacteria following CMTX-101 treatment are sensitized to the actions of many antibacterial agents, allowing the increased effectiveness of first-line antibiotics and the reservation of second- and third-line antibiotics for the most serious infections with determined genotypic resistance. Hence, effective killing with first-line antibiotics used in combination with CMTX-101 may reduce the probability of further development and spread of bacterial resistance.

## 7. Conclusions

Previously, Watnick and Kolter drew an analogy between biofilms and cities; bacteria within biofilms settle with a selective division of labor that is regulated through communication, store energy, and transfer genetic material for the “good of the many” [69]. This characterization was further expanded when Flemming et al. referred to the EPS as the “house of the biofilm cells” due to its role in determining the living conditions within the biofilm [26]. Here, we describe the effect of rapid biofilm collapse following treatment with anti-DNABII antibodies and the resulting transient increased susceptibility of resident bacteria to antibiotics. By envisioning the protective properties of a biofilm using the metaphor of a fortified medieval castle, we liken this phenomenon to the crumbling of the castle walls without giving the resident knights sufficient time to put on their armor and go into battle to defend the community. With the knights not fully prepared to fight, the entire community can be more easily overwhelmed by opposing forces.

We recognize that CMTX-101 may have some limitations in the clinical armamentarium. As CMTX-101 is currently designated for intravenous administration, clinical use would require hospital facilities or outpatient infusion. For certain clinical indications (e.g., prosthetic joint infections), CMTX-101 may have limited access or distribution to the site of infection following parenteral administration, thereby prompting the need for a different route of administration, such as direct injection at the infection site. Additionally, biofilm disruption may not necessarily overcome any genetic resistance mechanisms to antibacterial agents; however, the increased sensitization phenomenon demonstrated for NRel bacteria may be sufficient to sensitize bacteria to one or more classes of antibiotics.

Recognizing these limitations, we feel that CMTX-101 represents a new weapon in the pharmacologic arsenal to “crumble the castle” and combat biofilm persistence and AMR development. With no new antibiotic classes developed in over 30 years, the next generation antibiotics are currently being reserved for use in only the most critical cases. CMTX-101 enables the use of “old” antibacterial agents, whose use is currently limited by AMR, while reducing the pressure to use “new” antibacterial agents as first- or second-line therapies. The benefit of CMTX-101 applies to many clinical indications resulting from bacterial infections by augmenting first-line antibiotic therapy effectiveness and increasing patient response. The pathogen-agnostic application of CMTX-101 has a strong potential to address the serious public health threat caused by AMR.

## Figures and Tables

**Table 1:** BLAST search results for pathogens of interest showing amino acid sequence homology (% identical) for DNABII proteins (IHF and HU) compared to the α- and β-tip chimer peptide [48] used to generate CMTX-101.

Pathogen	IHF		HU	
	α-Tip 20-Mer Peptide (% Identical)	β-Tip 20-Mer Peptide (% Identical)	α-Tip 20-Mer Peptide (% Identical)	β-Tip 20-Mer Peptide (% Identical)
WHO Priority 1: Critical				
*Acinetobacter baumannii*	90	85	80	80
*Pseudomonas aeruginosa*	85	90	75	78
*Enterobacteriaceae (Serratia marcescens)*	75	80	47	79
WHO Priority 2: High				
*Enterococcus faecium*	45 *	50 *	47	45
*Staphylococcus aureus*	NA	NA	78	67
*Helicobacter pylori* ^‡^	ND	ND	NS ^‡^	NS ^‡^
*Campylobacter* spp. *(C. jejuni)*	75	80	47	NS
*Salmonella* spp. *(S. enterica)*	75	80	47	45
*Neisseria gonorrhoeae*	80	65	45	56
WHO Priority 3: Medium				
*Streptococcus pneumoniae*	NA	NA	78	86
*Haemophilus influenzae*	100	100	47	100
*Shigella* spp*. (S. dysenteriae)*	90	80	47	45
Other				
*Burkholderia cenocepacia*	85	80	42	50
*Escherichia coli*	95	80	75	80
*Klebsiella pneumoniae*	95	80	80	80
*Enterococcus faecalis*	75	80	47	45
*Mycobacterium tuberculosis*	75	75	47	45
*Aggregatibacter actinomycetemcomitans*	90	80	90	80
*Moraxella catarrhalis*	80	85	53	55

NA—not applicable, only HU present; NS—no significant similarity found; ND—not determined. * Based on comparison to *E. faecium* IHF (GenBank: PWQ88795.1. ^‡^ Based on comparison to *H. pylori* HU family DNA-binding protein (NCBI Reference Sequence: WP_199498384.1).

**Table 2:** Bacterial species and strains tested to date in either in vitro, ex vivo, or in vivo models, including ESKAPEE bacteria (*E. faecium, S. aureus, K. pneumoniae, A. baumannii, P. aeruginosa, Enterobacter* spp., and *E. coli*).

Biofilm	In Vitro	Ex Vivo	In Vivo	Source [Reference]
Nontypeable *Haemophilus influenzae*	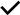	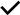	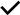	Clinical isolate [49,51,61]
*Streptococcus pneumoniae*	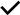			Strain 1121 [45,61]
*Moraxella catarrhalis*	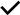			Strain 7169 [50]
*Streptococcus mutans*	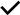			Strain UA159 [45]
*Staphylococcus epidermidis*	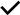			Clinical strain 1618 [45]
*Neisseria gonorrhoeae*	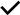			Strain 1291 [45]
*Burkholderia cenocepacia*	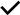			Multiple strains [39,50,61]
*Aggregatibacter actinomycetemcomitans*	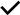		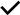	Clinical strain D7S-1 [58]
*Porphyromonas gingivalis*	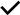			Strain 381 [57]
*Streptococcus gordonii*	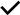			Chalis CH1 [57]
*Streptococcus mitis*	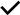			ATCC 33399 [56]
*Streptococcus cristatus*	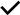			ATCC 49999 [56]
*Salmonella typhi*	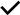			Multiple strains [62]
*Streptococcus oralis*	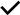			ATCC 10557 [56]
*Enterococcus faecium*	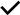			Unpublished data
*Staphylococcus aureus (MRSA, MSSA)*	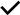		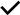	ATCC 29213 (MSSA) [45,61]; Unpublished data (MRSA)
*Klebsiella pneumoniae*	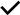			Unpublished data
*Acinetobacter baumannii*	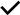			Strain 17978 [61]
*Pseudomonas aeruginosa*	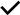		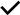	ATCC 27853 [45]; Strain 142-1 [61];Unpublished data
*Enterobacter* spp.	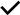			Unpublished data
*Escherichia coli*	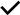			Strain MG1655; Uropathogenic strain UTT89 [45]
*Mycobacterium smegmatis*, *M. abscessus*	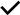			Unpublished data
Polymicrobial		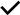		Cystic Fibrosis sputum [46] Cesarean section wound [60]

## Data Availability

Not applicable.
